# Ocular Surface and Tear Film Changes in Older Women Working with Computers

**DOI:** 10.1155/2015/467039

**Published:** 2015-10-18

**Authors:** Alfredo Ribelles, Carmen Galbis-Estrada, Maria A. Parras, Bárbara Vivar-Llopis, Carla Marco-Ramírez, Manuel Diaz-Llopis

**Affiliations:** ^1^Occupational Medical Services, General Treasury of the Social Security Administration, 46001 Valencia, Spain; ^2^Ophthalmology Research Unit, Faculty of Medicine and Odontology, University of Valencia, 46007 Valencia, Spain

## Abstract

The aim of this work is to investigate changes in the ocular surface (OS) and tear film (TF) by means of questionnaire-based subjective symptoms, TF break-up time, Schirmer test, and TF analysis in women working with computers and to analyze the effects of the oral supplementation with antioxidants/omega 3 fatty acids (A/*ω*3) in the OS outcomes. Women aged 40–65 years (*n* = 148) were recruited at the Administrative Offices of Valencia (Spain) and distributed into two age groups, 40–52 years (AGE1; *n* = 87) and 53–65 years (AGE2; *n* = 61), and then subdivided according to being (or not) computer users (CUG; NCUG) during the workday. Homogeneous subgroups were randomly assigned (or not) to the daily intake of three pills of A/*ω*3 for three months. At baseline and at the end of follow-up, personalized interviews and ocular examination were done. Reflex tear samples were collected from the inferior meniscus and processed for a multiplexed particle-based flow cytometry assay to measure proinflammatory molecules. Statistics were performed using the SPSS 15.0 program. The OS pathology was clinically evident in the AGE1-CUG (33%) versus the AGE2-CUG (64%) of women. Significantly higher interleukins-1*β* and -6 tear levels were found in the AGE1 versus the AGE2 women employees (*P* = 0.006 and *P* = 0.001, resp.), as well as in the CUG versus the NCUG (*P* = 0.001 and *P* = 0.000, resp.). Supplementation with A/*ω*3 positively influenced the OS pathology as manifested by the amelioration of the clinical signs/symptoms related to computer uses. Strategies involving a safe environment and oral micronutrient supplements may be managed within eye-care standards in older women.

## 1. Introduction

The ocular surface (OS) constituents (cornea, conjunctiva, eyelids, and tear film) and the lacrimal and accessory glands with the corresponding drainage system are essential for vision. When they fail in preserving the integrity of the ocular surface, tear film impairment and ocular surface pathologies appear as dry eyes (DEs) [1–4]. This disorder usually affects elder people aged 50 years or more [5–7] particularly women, with an estimated 3.23 million American women experiencing DEs [8]. Strong evidence suggests that having DEs is a multifactorial process in which multiple risk factors such as genetic, age, sex, nutrition, environmental conditions, lifestyle, working characteristics, immune situation, hormonal status, and medications contribute to alter the morphology and function of the ocular surface constituents, leading to the pathological manifestations of DEs [1, 3, 9]. Environmental conditions play a relevant role in the apparition and progression of disease. Among the causes related to DEs are the following: temperature, humidity, wind, fumes, pollution, air speed, CO_2_ concentration, and light intensity [3, 9]. Currently, there are two principal types, the clinical form that results from gland dysfunction (aqueous deficient) and the clinical form induced by the meibomian gland disorder (evaporative). The affected patients, mainly women, commonly display a mixture of both DEs types, independently of the etiology, with the most frequent symptoms being dryness, burning, stinging, grittiness, and foreign body sensation that usually are accompanied by visual impairment and loss of quality of life [1–6, 9].

What are the most important causes for eye and vision changes with aging? Firstly, in our 40s, the presbyopia appears and some refractive errors worsen. This is the time for being aware of increased risk for hypertension eye pressure, DEs, and/or computer use-derived problems. Next, in the 50s, there is an increased risk for cataracts, macular degeneration, DEs in women after menopause, and vascular disorders affecting the eyes and vision. Then, in the 60s and more, there is an increased risk for the most common age-related eye diseases including glaucoma, cataracts, DEs, retinal vascular occlusion, neurodegenerative disorders, and/or systemic diseases and comorbidities, with the age-related macular degeneration being the major ophthalmic problem in this decade.

It has been well documented that DEs prevalence rises exponentially with aging, and as older populations grow the disease becomes a much more important health issue and socioeconomic problem [1, 10, 11]. Furthermore, it has been reported that 1/10 women in their seventies and 1/20 women over the age of 50 complain of one or more dry eye symptoms in the United States [8]. A recent report revisited the impact of androgens on the morphology and function of the meibomian and lacrimal glands suggesting that androgen deficiency is associated with the etiopathogenic mechanisms of DEs [12]. However, other reports regarding the influence of estrogen and progesterone on the OS are contradictory. It has been described that testosterone regulates the expression of multitude of genes in the lacrimal/meibomian glands in the ovariectomized mice model [13]. Oprea et al. [14] suggested that optimal androgen levels are essential for lacrimal gland function and that prolactin and estrogens also play relevant roles on this activity.

Office employees often work in front of a computer. Adverse effects of such exposure have been referred to as computer-vision syndrome (CVS) that is a disorder resulting from focusing the eyes on a computer during noninterrupted time periods [15–18]. In this context, computers (Cs) users complain of a variety of ocular signs and symptoms such as itchiness, soreness, foreign body sensation, irritation, photophobia, redness, eye strain, tired eyes, blurred vision, double vision, and headache [19–22]. Ergonomic and ophthalmologic characteristics in the Cs-exposed individuals, including ameliorating symptoms by changing the computer location and/or the display features, the body position, and the light incidence as well as using eye solutions to improve spontaneous blinking and ocular good feeling (tested during work at video display terminals and during inactivity), were reported [19, 22].

Several etiopathogenic theories for DEs have arisen, with the autoimmune, caloric restriction or oxidative stress processes among them [23–27]. In this scenario, immune system involvement in OS pathologies has been documented in humans and animal models [28–31]. The OS alterations induce inflammation that leads in part to the development of an epithelial disorder and sensations of irritation [30–32]. Numerous immune response biomarkers, including proinflammatory cytokines and chemokines, have been identified in tears and the conjunctival and corneal epithelia of DEs patients [33–36]. These immune response mediators can trigger an inflammatory cascade on the ocular surface and subsequent signs and symptoms. However, the switch clock and specific mechanisms by which dryness and irritation stimulate chronic inflammation in the ocular surface have not yet been elucidated. Moreover, the pathogenic processes for OSDs in adults exposed to visualization screens in the office have not been fully evaluated.

Age-related ophthalmic pathologies are major causes of vision impairment and blindness worldwide. The antioxidant vitamins and essential polyunsaturated fatty acids (including *ω*3 and *ω*6) must be taken in the diet daily to meet physiological needs. However, epidemiological studies in well-nourished western populations suggested a role for nutritional supplements in delaying the onset of these disorders, but it is not yet possible to conclude that oral supplements can prevent cataracts, glaucoma, or macular degeneration [37–39]. In the case of *ω*3 fatty acids, these cannot be synthesized in sufficient amounts in the body, and deficiencies (that can worsen with aging) may cause well-defined symptoms [40, 41]. A recent report on dietary intake of *ω*3/*ω*6 from the International Society for the Study of Fatty Acids and Lipids specifically recommended the following proportions: linoleic acid intake (2 energy %) and *α*-linolenic acid intake (0.7 energy %), but a minimum intake of eicosapentaenoic acid and docosahexaenoic acid combined (500 mg/d) for optimum cardiovascular health [42]. The latest evidence has shown that the appropriate *ω*3 intake reduces the expression of inflammatory biomarkers in humans [43]. These findings propose a possible protective function of *ω*3 supplementation against inflammation.

To our knowledge, in addition to the aging process, the role of risk factors involved in the development/progression of DEs in women employees, particularly the exposure to Cs and the implications of the inflammatory mediators in this process, has not been fully investigated. In the present study we also deal with a more definitive evaluation of the effects of the oral supplementation with A/*ω*3 in the aging women employees suffering from DEs.

## 2. Material and Methods

### 2.1. Study Design

The present study is a prospective, randomized, open-label work that was approved by the Institutional Review Board of the University Hospital Dr. Peset (Valencia, Spain) (Ref: CEIC 2013). The authors carefully observed all tenets of the Declaration of Helsinki for the protection of human subjects in medical research.

Our main hypothesis was that older women employees working with Cs during the workday may be vulnerable to DEs compared to the nonuser women. We also hypothesized that exacerbation of inflammation in the OS constituents may be involved in the pathogenic mechanisms of DEs. Moreover, we wanted to test whether or not an appropriate supplementation with A/*ω*3 may ameliorate the clinical manifestations and personal impressions related to DEs and also to reduce the tear expression of inflammation biomarkers in the aging women employees. The present study was designed to provide information on these issues.

### 2.2. Participants and Study Groups

Under the main criteria for inclusion and exclusion (Table 1), we recruited 148 women aged 40–65 years. Participants were selected consecutively from those attending the occupational medical services of the general Treasury Administration Offices of the Spanish government in Valencia (Spain), between the years 2012 and 2013. All suitable participants signed an informed consent.

Women participants were advised to discontinue for at least 3 months the use of nutritional supplements, systemic antihistaminics, and any treatment related to DEs. Those participants using contact lenses or suffering obvious eye and adnexa infection or notable eyelid inflammation were excluded from this study.

In parallel to supplementation, a dietary control of all participant women was performed to assess possible interferences of diet on the results of this study.

**Figure 1:**
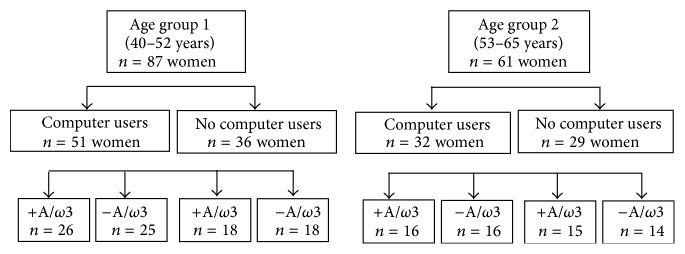
Classification of participants according to the main groups and subgroups.

Participants were assigned to one of the following main groups as reflected in Figure 1: participants aged 40–52 years (AGE1 group; *n* = 87) and participants aged 53–65 years (AGE2 group; *n* = 61). Moreover, in each of these groups the women employees were classified as Cs users (CUG; *n* = 83) and nonusers (NCUG; *n* = 65). Homogeneously, employees from each group were randomly assigned (or not) to the daily intake of three pills containing A/*ω*3 for three months. Thus, these latter participants were alternatively classified as +A/*ω*3 (*n* = 75) and −A/*ω*3 (*n* = 73). The A-*ω*3 formulation used was Brudysec 1.5 (Brudylab, Barcelona, Spain), each pill containing vitamin A (133 *μ*g), vitamin C (26.7 mg), vitamin E (4 mg), tyrosine (10.8 mg), cysteine (5.83 mg), glutathione (2 mg), zinc (1.6 mg), copper (0.16 mg), manganese (0.33 mg), selenium (9.17 *μ*g), docosahexaenoic acid (350 mg), eicosapentaenoic acid (42.5 mg), and docosapentaenoic acid (30 mg). The A-*ω*3 formulation (Brudysec 1.5) was produced by Brudylab (Barcelona, Spain) who gently donated the capsules for this study. Compliance with the oral supplement by the participants was one important point of this study, necessary to emphasize the effectiveness of the components and the reliability of the study data. After the basal appointment, women participants were visited every month during the study course (3 months) for recording incidences and feelings regarding the eyes, the job, and the A/*ω*3 intake. Each patient underwent 1 basal screening and 3 visits in this study.

### 2.3. Proceedings

Interviews and ophthalmological examination were performed for all study subjects; especial importance was placed on the signs and symptoms of DEs and the participant subjective sensations. The OS disorder index (OSDI (Allergan Inc., Irvine, California, has the copyright)) questionnaire was carried out for all participants for differentiating those normal, mild, moderate, or severe DEs, as done before [33, 44]. The overall OSDI score delineated the OS from normal (0–12 points) and mild level of disorder (13–22 points) and moderate disorder (23–32 points) to severe stage of disease (33–100 points). The OSDI questionnaire was done during the medical appointments.

The effectiveness of A/*ω*3 was evaluated by studying the clinical and biochemical changes, as well as the subjective impressions of the participants through the 3 months of follow-up.

All participants were examined by the Occupational Medical Services staff at the general Treasury Administration Offices with the help of one ophthalmologist. Examinations included the Schirmer test to quantify tear secretion, blinking frequency (near), ocular surface inspection, and corneal characteristics under fluorescein staining. Schirmer test was performed by placing a small strip of filter paper in the lower eyelid (inside), without previous anesthetic drop instillation, to observe the amount of wetting the strip during 5 min. The blinking frequency was determined by recording the spontaneous number of times of closing eyelids that occur in 1 min, with the participant seated in front of the computer station, under working conditions.

First ocular data considered for the DEs diagnosis and the effectiveness of the A-*ω*3 formulation were the Schirmer test and blinking frequency, and secondary outcome measures were the DEs symptoms and subjective sensations.

Special attention was paid to the workplace conditions in the office to better understand ocular surface changes in the employees. Data were obtained from the following homologized systems: heat stress monitor, indoor air quality (Microtherm IAQPROBE DAE 504002), light (luxometer Gossen Mavolux 5032 C/B n° serie 0C60759), and CO_2_ concentration analyzer Ex 2000 Oldham/CO_2_.

**Figure 2:**
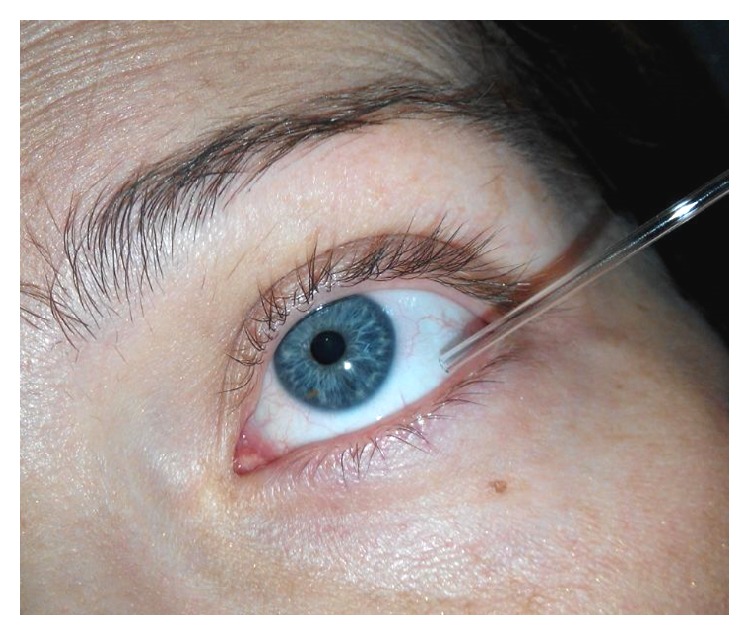
Collecting method of reflex tears by a Pasteur micropipette.

Next, we collected tears samples from all women participants to be analyzed through biochemical approaches. The gentle rubbing method was used to obtain reflex tears from the inferior meniscus of both eyes of our participants, by means of a micro Pasteur, as previously described [35, 36, 45] and shown in Figure 2. Collected tear samples from both eyes were immediately deposited in micro Eppendorfs to be frozen and stored at −80°C until assaying a specific set of inflammatory mediators. The human panel of cytokines/chemokines that was assayed in this study was composed of the following interleukins (IL): IL-1*β*, IL2, IL4, IL5, IL6, IL7, IL8, IL10, and IL12; tumor necrosis factor-alpha (TNF-*α*); vascular endothelial growth factor (VEGF); granulocyte-macrophage-colony stimulating factor (GM-CSF); and interferon-gamma (IF-*γ*). The analyses were performed by the Luminex R-200 multiplex system (Luminex, Austin, TX, USA), as reported before [33, 35, 36]. Polystyrene beads coupled covalently to specific antibodies (cytokines/chemokines) were prepared to react with an approximate amount of 20 *μ*L of each tear sample (which contains an unknown amount of these molecules), or with a standard solution (having a known amount of molecules), at room temperature for 1 hour. For describing briefly the protocol, a series of washes (to remove unbound proteins) were done. Biotinylated detection antibody specific for a different epitope on the cytokine was added to the beads and incubated at room temperature for 30 min. Streptavidin-phycoerythrin (which binds to the biotinylated detection antibodies) was used to detect the reaction mixture. Next, the flow-based Bio-Plex (Bio-Rad Laboratories, Hercules, CA, USA) suspension array system was used to identify and quantify each antigen-antibody reaction. The assayed set of inflammatory molecules were identified by means of a method of bead color and fluorescence, using fluorescently labeled reporter molecules associated with each target protein. Unknown cytokine/chemokine levels were calculated automatically by the Bio-Plex Manager software (Bio-Rad Laboratories) by using a standard curve derived from a recombinant cytokine standard. Tear levels of the cytokine/chemokines were corrected for the initial total protein concentration and finally expressed as mean ± SD of three independent measurements.

Data were recorded in a designed Excel sheet (Microsoft Corporation, Redmond, WA, USA) and reflected as the mean ± SD. A parametric test (*t*-student) was used for comparing two independent sample groups by means of the SPSS software (IBM Corporation, Armonk, NY, USA). Results were statistically analyzed to detect differences between the two groups, and *P* < 0.05 was considered statistically significant.

## 3. Results

### 3.1. Demographics and Workplace Characteristics

Mean age of all women employees was 54 ± 8.5 years; among them, the AGE1 group (aged 40–52 years) displayed a median age of 46 ± 6 years, with 65% of these women being menopausal, whereas the AGE2 group (composed of the women aged 53–65 years) had a median age of 60 ± 4 years, with 100% of them being menopausal. Furthermore, mean age of the CUG was 53 ± 5 years versus 50 ± 10 years of the NCUG.

An important point to consider was the average duration of Cs uses during the office workday among the women employees, and it was 4.5 ± 2 hours. It has to be emphasized that the type of screen and the Cs were similar for all participants, and the clinical probes and tear collection were performed at the end of the daytime in all participants.

Moreover, all study participants were exposed to the same controlled environment during the working time. The environmental conditions were evaluated periodically by means of the workplace analyses (Table 2).

### 3.2. Evaluation of the Ocular Surface Status

A clinician global impression as well as a participant global self-assessment was the endpoint to estimate the OS status that was completed by the OSDI questionnaire scores. The Cs user women from the AGE1 and AGE2 groups complained of one or more DEs signs/symptoms of the following: itchiness, soreness, irritation, foreign body sensation, photophobia, redness, eye strain, tired eyes, eye pain, blurred vision, vision loss, or headache associated with eye pain.

The overall OSDI score delineated the OS severity. It was diagnosed that 33% of the AGE1 and 64% of the AGE2 Cs users had mild or mild-to-moderate DEs, as confirmed by the anatomic and functional eye probes. Furthermore, most of these women participants (89%) utilized eye drops and none of them had severe dryness or Sjögren syndrome.

As shown in Figure 2, the Schirmer test scores (by wetting the paper strip during 5 min) were significantly lower in the AGE1-CUG and AGE2-CUG groups than in the NCUG of women employees (*P* = 0.0002 and *P* = 0.0000, resp.). These data reflect the altered tear film in the women using the Cs during the working time (Figure 2).

The blinking frequency (near) for the right and left eyelid values were combined and analyzed as a function of age and the results showed lower frequency in the AGE1-CUG and AGE2-CUG (9.5 ± 3.81/5.77 ± 2.27 blinking per 1 min, resp.) than in the NCUG (14.55 ± 6.50/9.61 ± 4.98/blinking per 1 min, resp.). Our results strongly suggest that there is a trend toward decreasing blink amplitude and peak velocity with age for spontaneous blinks. Furthermore, the blinking process is altered by the exposure to the visualization screen in women employees, compared to the nonusers (*P* = 0.000).

### 3.3. Multiplex Analysis of Inflammatory Molecules in Tears

With the assayed amounts of tears utilized in the present work (mean 14 ± 8 mL) it was permitted to detect the majority of molecules related to inflammation (as in the human cytokine panel utilized herein) in 92% of the samples. Polystyrene beads coupled covalently to specifically directed antibodies (cytokines/chemokines) were allowed to react with each tear sample containing an unknown amount of them, or with a standard solution containing a known amount of these molecules, at room temperature for 1 hour, following the manufacturer's instructions. Detection data of the inflammation molecules from the tear samples of the women employees are summarized in Table 3 and expressed in picograms/*μ*L. The following molecules showed very low or undetectable levels in the tear samples: IL4, IL8, and VEGF, and the results were excluded from Table 3.

**Figure 3:**
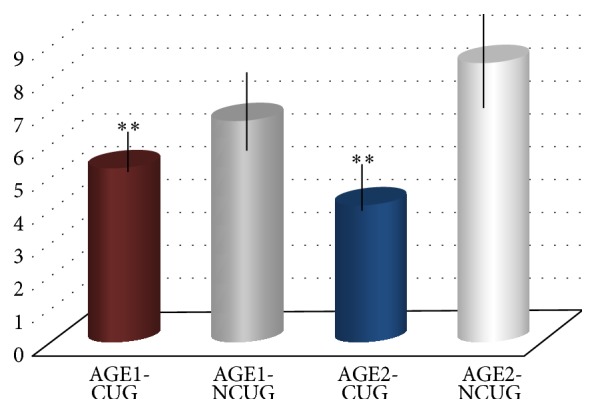
The Schirmer test scores in the age groups of women employees and in relation to being exposed or nonexposed to computer screens during the workday. Data are mean ± SD for all participants in each group.

Data comparison for the AGE1 and the AGE2 women employees revealed that the IL-1*β* and IL6 tear levels were significantly higher (a twofold increase) in the older women compared to the younger employees (Figure 3).

**Figure 4:**
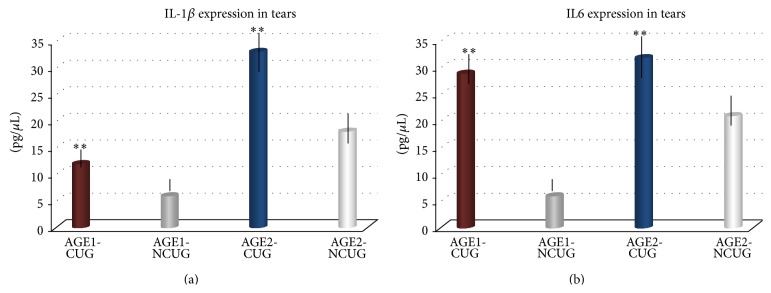
(a) Data comparison of the multiplex tear samples analyses for the two main age groups of women employees exposed and nonexposed to computer screens regarding the IL-1*β* tear expression levels. (b) Determination of IL6 expression in tears compared to the computer screen exposures in the age study groups of women participants. Bars, mean ± SD. Significance levels were taken at ^∗^ *P* < 0.01; ^∗∗^ *P* < 0.001.

In relation to the Cs uses, when comparing the AGE1-CUG versus the AGE1-NCUG and the AGE2-CUG versus the AGE2-NCUG the results also showed statistically significant differences in the tear expression of the assayed kit of cytokines/chemokines, with the most relevant concentrations of proinflammatory mediators pertaining to the IL-1*β* and IL6, as reflected in Figure 4.

### 3.4. Influence of the Oral Supplementation with Antioxidants and Omega 3 Fatty Acids

Average amount of the collected tear samples was 24.9 ± 6.8 *μ*L from the controls versus 15.6 ± 4.8 *μ*L in the DEs group. This latter augmented noticeably after supplementation (about 25%) in the AGE2 group and the CUG of women employees that were taking the A-*ω*3 supplement as compared to those not taking the A-*ω*3 pills.

**Figure 5:**
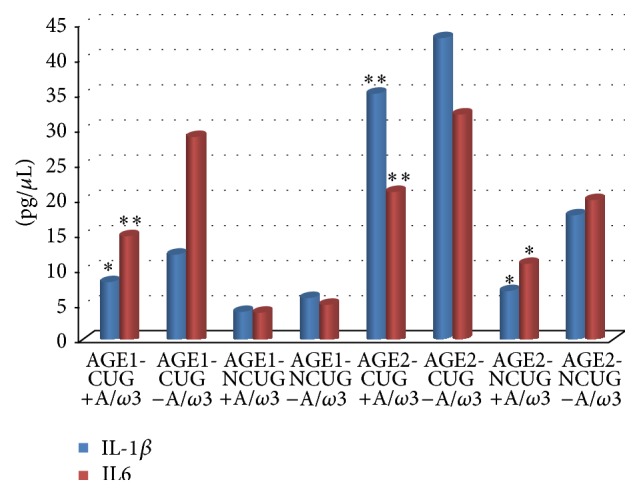
Expression levels of main inflammatory biomarkers in tears from the supplemented and the nonsupplemented women employees, according to the main age groups. Comparative analysis between groups (significance level) ^∗^ *P* < 0.01; ^∗∗^ *P* < 0.001.

A significant reduction in the expression levels of the inflammation biomarkers was detected in the AGE1-CUG and AGE2-CUG supplemented groups in contrast to the nonsupplemented women employees. A more precise analysis strongly indicated that the IL-1*β* and IL6 were the most significantly reduced proinflammatory biomarkers in the A/*ω*3 supplemented study subjects (Figure 5).

Up to 70% of the AGE1-CUG and AGE2-CUG women taking the supplement pills according to the prescribed doses (+A/*ω*3) significantly improved their subjective/objective DEs-associated manifestations at the end of the study, as compared to the participants not taking the oral supplementation (−A/*ω*3).

No adverse effects were recorded in relation to the oral intake of this supplementation in the corresponding subgroups.

## 4. Discussion

### 4.1. Age-Related Ocular Surface Disorders

Visual impairment in adults and older people is a major health problem worldwide. Age is a risk factor for OS disorders, especially in women [5, 6, 10, 11, 38, 46]. The DEs are characterized by ocular surface damage, reduced tear film stability, and tear hyperosmolarity accompanied by signs and symptoms [1, 2, 4]. Inflammatory components are also considered within the DEs [28–32, 44], which is also an important point of the present work.

In this study we have evaluated the OS status in a sample of healthy adult women employees that were Cs users during the working time, following an age-related fashion. Demographic, ophthalmologic, and molecular data were obtained from all participants in the study and the integration of these data allowed us to better evaluate the incidence and severity of age-related DEs in our women employees. In fact, an abnormally low Schirmer test and reduced spontaneous blinking frequency were found in the older women compared to the younger employees, in agreement with previous reports [16, 20, 46, 47]. These data regarding the high DEs prevalence in relation to aging strongly agree with similar reports [5–7, 10, 11, 18, 46]. In a recent review it was confirmed that conditions predisposing older adults to DEs include systemic and topical medications, eyelid laxity, menopause, and chronic systemic inflammation [4, 7, 10, 11, 47]. In this context, early detection and adequate management of DEs in the older Cs user women may help in preventing ocular surface complications such as corneal ulcers and scarring leading to visual disability.

### 4.2. Computer Screens Exposures in the Women Employees

It has been reported that in adult employees the Cs use and/or the body position may influence the visual performance and the eye comfort [15, 22]. Our representative population of Cs user women employees had a mean of 4.5 hours of exposure, and their symptoms were similar to those reported in surveys of video terminal users during the working time [19–22].

To fully evaluate the risk factors that may compromise the OS integrity in the employees, we took advantage of the controlled environmental conditions of the workplace provided to our study employees. Individuals are vulnerable to adverse environments that may increase tear evaporation and decrease goblet cell density and acquired ocular surface pathology. Lifestyle factors contributing to DEs include working in a dry atmosphere; looking at visualization screens or reading without blinking frequently; treatments for allergy; use of diuretics, beta-blockers, antispasmodics, birth control pills, and other medications; diets that provide insufficient water or essential fatty acids; autoimmune disorders (arthritis, lupus erythematosus); and menopause [1, 3, 6, 9]. No systemic chronic disorders and no special local or systemic treatments and/or particular exposures to external or internal damaging agents were recorded. Therefore, our controlled parameters in the office allow us to exclude these factors for DEs, pointing to both the age and gender, as well as the Cs uses as major risk factors for OS pathologies in our middle-age and older population of administrative women office employees.

The incidence of DEs that was diagnosed during the present study in the Cs user employees was noticeable, with an important prevalence in the group of older women (33% of the AGE1 and 64% of the AGE2). It has to be emphasized that 100% of the older women were menopausal, with the hormonal disbalance (testosterone, progesterone, and estrogens), independently of the hormone replacement therapy, being a relevant factor in the initiation and progression of DEs. [12–15, 48]. Furthermore, decreased Schirmer test scores and blinking frequency were seen in the CUG as compared to the NCUG of women employees, as depicted in similar works [15, 16, 19, 22].

We also considered that Cs uses may extend beyond work activities and into leisure time. When asked about this, women of the CUG confirmed only low utilization of video games, internet, and social networks. Furthermore, given that the environmental conditions in the office were periodically recorded and accordant with a healthy workplace (see Table 2) and that no participants had systemic disease, aggressive treatment, or Sjögren syndrome, the fact of using Cs during the workday appears to be a major risk factor for the development and progression of DEs in our women employees. In spite of this, current information is insufficient to completely understand the basic cellular and molecular mechanisms underlying DEs.

### 4.3. Inflammatory Mediators in Tears of the Women Employees

The role of inflammation in the pathogenic mechanisms of DEs has also been investigated in the present work. Previous reports demonstrated an altered tear composition in DEs [2, 15, 17, 23–27, 45], including in air-controlled conditions for the study participants [49], as in the present work. A common underlying cytokine/chemokine-mediated inflammatory disorder in all ocular surface pathologies has been suggested, independently of the etiology [28–36, 44]. Results from the quantification of tear components related to the immune system among the study participants showed significant differences between groups and subgroups. In fact, the set of cytokines/chemokines assayed herein showed a differential expression profile regarding age (as shown in Table 3 and Figure 3). The most relevant differences were detected in relation to IL-1*β* and IL6, these two cytokines being important proinflammatory mediators involved in DEs [31–33, 44]. Moreover, the IL-1*β* and IL6 also showed significantly higher levels in the CUG as compared to the NCUG of women employees, reflecting a relevant inflammatory background in tears from these computer user patients. Interestingly, the increased cytokines in tears of the AGE2-CUG versus the AGE1-CUG of women employees strongly correlated with clinical DEs parameters, with results being in agreement with previous reports [31, 33–36]. Outstanding statements regarding inflammation and DEs from the Cullen Symposium on Corneal and OS Inflammation (Baylor College of Medicine, Houston, Texas, USA; 2005) strengthen the main results of the present work. According to this, cytokines produced by activated T cells increase the immune response by mediating adhesion molecules expression from the conjunctival blood vessels [41]. With the data provided by the tear analysis performed during the present work we may also contribute to rising inflammation as a milestone for the development and progression of DEs in middle-age and older employees exposed to visualization screens in the office. The host defense against chitin-containing pathogens includes production of chitinases. In this context, Musumeci et al. [47] and Bucolo et al. [50] have studied the role of AMCase in relation to OSD suggesting that chitinases may be important mediators of the inflammatory processes, constituting a potential diagnostic and therapeutic target in these pathologies.

### 4.4. Effect of a Combination of Antioxidants and Omega 3 Fatty Acids in the Ocular Surface Disorders

At this point the question as to whether a combined formulation of A/*ω*3 may influence the evolution of DEs signs and symptoms in the affected women employees arises. It has been described that antioxidant supplements may help in counteracting the oxidative stress generated in the anterior eye segment pathologies [24, 27, 37, 38]. In addition, specific *ω*3 metabolic by-products may play an essential role in modulating the inflammatory response in health and disease [40]. Main results of our study are the significantly lower expression levels of the inflammatory molecules found in the CUg +A/*ω*3, as compared to the CUG −A/*ω*3, in agreement with previous reports [51–53]. Likewise, recent research from our group [36, 37] described the fact that a combination of A/*ω*3 improved cytokine/chemokine expression levels in tears of the affected patients as well as the subjective and objective DEs manifestations. In the present work, global amelioration in the clinical signs and symptoms was registered among the CUG +A/*ω*3 compared to the −A/*ω*3, at the end of follow-up. Up to 70% of ocular signs (dryness, photophobia, eye heaviness, burning sensations, and blurred vision) displayed a noticeable improvement in the supplemented subgroups of women employees.

A limitation of this study is the absence of a placebo group to the A/*ω*3 oral supplementation participants. This was an open-label study with a potential bias, but it was reduced with the use of randomization and the utilization in parallel with the nonsupplemented subgroups.

## 5. Conclusions

Considering these findings, we may suggest that a specific appropriate combination of A-*ω*3, as in the present work, may benefit the OS integrity in women employees that were Cs users during the working time. Further research will indicate whether the supplement is also effective (or not) in redressing the OS damage. Moreover, cytokine/chemokine expression and availability in tear samples can be helpful biomarkers for diagnosing women at risk of DEs and visual impairment in relation to computer work.

Given the estimated number of the population at risk of DEs due to the Cs uses during the workday, the challenge that lies ahead is of real impact and requires screening campaigns among adult employees, mainly those women at age of 50+ and working with Cs, for detecting DEs cases that remain occult. Nutraceutics with an appropriate combined formulation of A-*ω*3 may help in managing DEs in women employees with daily computer work.

## Figures and Tables

**Table 1:** Inclusion and exclusion criteria for the study participants.

Inclusion criteria	Exclusion criteria
Women	Man
40–65 years	<40 or >65 years
Employees usually working with computers (CUG)	No computer user employees (NCUG)
With or without previous diagnosis of mild DEs	Eye disease, contact lenses, laser therapy, or ophthalmic surgery within the previous six months
Agreement to participate and collaborate with the study	Neurodegenerative disease or aggressive treatments that may interfere with the study
	Taking supplemental antioxidants or essential fatty acid supplements
	Declined collaboration in the study or had a disabling disorder (established or being monitored)

**Table 2:** Analysis of the environmental conditions in the work place.

Environmental parameters	Data
Light intensity (lux)	500
Relative humidity (%)	32.673 ± 5.13
CO_2_ (ppm)	2370.71 ± 646.89
Air speed (m/seg)	0.11 ± 0.031
CO (ppm)	0
Dry temperature (°C)	24.56 ± 0.60

**Table 3:** Expression levels of the inflammatory molecules in tears from the women participants as expressed in picograms per microliter. These data are examined in greater detail in Figures 4 and 5.

	GM-CSF	IL2	IL-1β	IL5	IL10	IL6	TNF-α	IFN-γ
AGE1-CUG	5.5 ± 4	1.3 ± 0.7	12.9 ± 14.06	4.8 ± 5.9	2.2 ± 1.67	29.1 ± 2	215.1 ± 29.4	285.3 ± 32.5
AGE1-NCUG	6.5 ± 3	0.9 ± 1.25	6.7 ± 3	3.9 ± 6.3	3.4 ± 2.32	5.8 ± 1.2	233.6 ± 30	301.3 ± 257
*P* value	0.86	0.09	0.000	0.42	0.17	0.000	0.21	0.66

AGE2-CUG	7.6 ± 3	1.5 ± 0.2	43.1 ± 4.2	4.1 ± 7	3.02 ± 2.53	32.4 ± 5.22	223.3 ± 25.4	298.1 ± 321.5
AGE2-NCUG	7.5 ± 2.15	1.2 ± 2.3	18.7 ± 3.05	3.1 ± 5.4	2.5 ± 3	20.7 ± 7.6	217.1 ± 28	312.2 ± 77
*P* value	0.34	0.65	0.007	0.26	0.54	0.000	0.13	0.58

## Abbreviations

A/*ω*3: Antioxidants and omega 3 fatty acids
+A/*ω*3: Oral supplemented group
−A/*ω*3: Nonsupplemented group
AGE1: Women aged 40–52 years
AGE2: Women aged 53–65 years
Cs: Computers
CUG: Computer users group
DEs: Dry eyes
IL: Interleukins
NCUG: No computer users group
OS: Ocular surface
SD: Standard deviation
VEGF: Vascular endothelial growth factor
TF: Tear film.
